# HBV and HCV genotypes distribution on the territory of Belarus

**DOI:** 10.1186/1742-4690-9-S1-P57

**Published:** 2012-05-25

**Authors:** EL Gasich, VF Eremin, SV Sasinovich, MG Tulinova

**Affiliations:** 1 Briem, Minsk, Belarus

## Materials and methods

236 HCV and 158 HBV plasma samples collected during 2004-2011 have been investigated. EIA, PCR, RT-PCR, sequencing, SeqScape, BioEdit, MEGA4.1, statistica v.6 software have been used.

## Results

Of 158 HBV HBsAg positive specimens 17 (10,8%) were from patients with acute hepatitis B and 141 (89,2%) from chronic hepatitis B. Among surveyed were 83 (43,6%) women and 92 (56,4%) men at the age from 15 till 90 years. Middle age has made 44,5±17,8 years. From 158 surveyed 11 persons have been HBV+HBC co-infected and 1 with HBV+HCV+HIV. HBV genotypes has been defined at 61 (34,9%) patient. The phylogenetic analysis of HBV preS fragment has shown that in 52 (85,3%) cases come to light D genotype (D1 – 32.9%; D2 - 26,2%, D3 – 26.2%), in 7 (11,5%) – A (A2) and in 2 (3,2%) – C (C2) genotype. Of 236 HCV infected patients prevailed 1b (53,8%, n=127) and 3a (28,8%, n=58) genotypes. At 30 (12.7%) cases have been revealed 1a genotype and 2a and 2b genotypes have been revealed in 8 (3,4%) and 3 (1,3%) cases accordingly.

## Conclusion

In the territory of Belarus a genetic variety of HBV and HCV genotypes, caused both circulation before the brought viruses, and new drifts from neighbouring countries, basically from Russia and Ukraine is observed.

